# Metabolic management of microenvironment acidity in glioblastoma

**DOI:** 10.3389/fonc.2022.968351

**Published:** 2022-08-17

**Authors:** Thomas N. Seyfried, Gabriel Arismendi-Morillo, Giulio Zuccoli, Derek C. Lee, Tomas Duraj, Ahmed M. Elsakka, Joseph C. Maroon, Purna Mukherjee, Linh Ta, Laura Shelton, Dominic D'Agostino, Michael Kiebish, Christos Chinopoulos

**Affiliations:** ^1^ Biology Department, Boston College, Chestnut Hill, MA, United States; ^2^ Instituto de Investigaciones Biológicas, Facultad de Medicina, Universidad del Zulia, Maracaibo, Venezuela; ^3^ The Program for the Study of Neurodevelopment in Rare Disorders (NDRD), University of Pittsburgh, Pittsburgh, PA, United States; ^4^ Faculty of Medicine, Institute for Applied Molecular Medicine (IMMA), CEU San Pablo University, Madrid, Spain; ^5^ Neuro Metabolism, Faculty of Medicine, Alexandria University, Alexandria, Egypt; ^6^ Department of Neurosurgery, University of Pittsburgh, Medical Center, Pittsburgh, PA, United States; ^7^ Matterworks, Somerville, MA, United States; ^8^ Department of Molecular Pharmacology and Physiology, University of South Florida, Tampa, FL, United States; ^9^ BERG LLC, Framingham, MA, United States; ^10^ Department of Medical Biochemistry, Semmelweis University, Budapest, Hungary

**Keywords:** glutaminolysis, glycolysis, fermentation, succinate, lactate, glutamate, ketogenic diet, ketogenic metabolic therapy

## Abstract

Glioblastoma (GBM), similar to most cancers, is dependent on fermentation metabolism for the synthesis of biomass and energy (ATP) regardless of the cellular or genetic heterogeneity seen within the tumor. The transition from respiration to fermentation arises from the documented defects in the number, the structure, and the function of mitochondria and mitochondrial-associated membranes in GBM tissue. Glucose and glutamine are the major fermentable fuels that drive GBM growth. The major waste products of GBM cell fermentation (lactic acid, glutamic acid, and succinic acid) will acidify the microenvironment and are largely responsible for drug resistance, enhanced invasion, immunosuppression, and metastasis. Besides surgical debulking, therapies used for GBM management (radiation, chemotherapy, and steroids) enhance microenvironment acidification and, although often providing a time-limited disease control, will thus favor tumor recurrence and complications. The simultaneous restriction of glucose and glutamine, while elevating non-fermentable, anti-inflammatory ketone bodies, can help restore the pH balance of the microenvironment while, at the same time, providing a non-toxic therapeutic strategy for killing most of the neoplastic cells.

## Introduction

**Figure 1:**
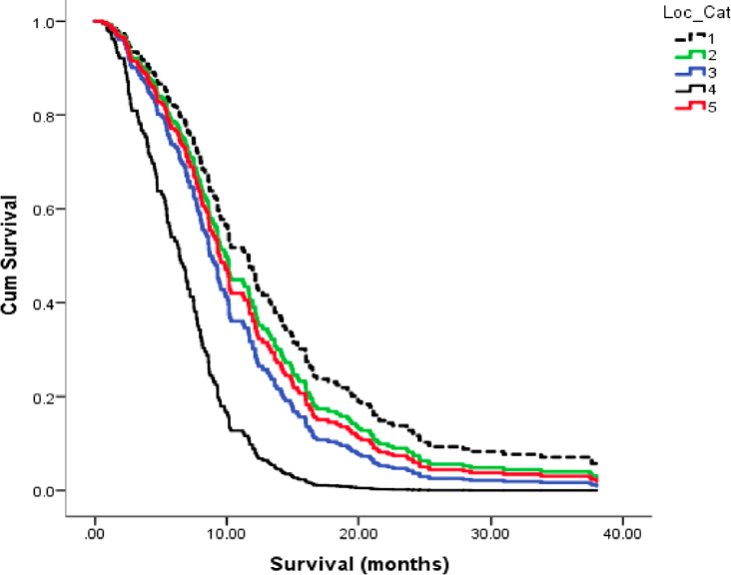
Kaplan-Meier plots for overall survival of GBM patients across five (1–5), Canadian surgical institutions. Each line represents patient survival for a particular institution as described (8). The GBM survival statistics recorded for these Canadian institutions are similar to those recorded in surgical institutions of other countries (2, 9, 10). These findings support the view of no major improvement of GBM patient survival in almost 100 years (8, 11). Image reproduced under a Creative Commons license from (8).

Glioblastoma (GBM) has among the highest mortality rates for primary brain tumors and remains largely unmanageable. Despite the hype surrounding newer therapies, median life expectancy following GBM diagnosis is only about 11-15 months with some large patient data bases reporting few survivors beyond 30 months (1–7). The poor overall GBM patient survival is also astonishingly consistent across many surgical institutions **Figure 1**. Although remarkable advances in science and technology have occurred over the last 100 years in Western societies, no significant advances have been made over this same period in improving survival for GBM patients (2, 7, 8). This abysmal lack of therapeutic progress can be due in large part to the inability to recognize GBM as a metabolic disorder (7, 12). Acidification of the GBM microenvironment arises as a consequence of the fermentation metabolism within the neoplastic tumor cells and is largely responsible for therapy failure. This review provides the evidence supporting this statement.

## Fermentation metabolism is responsible for GBM growth

**Figure 2:**
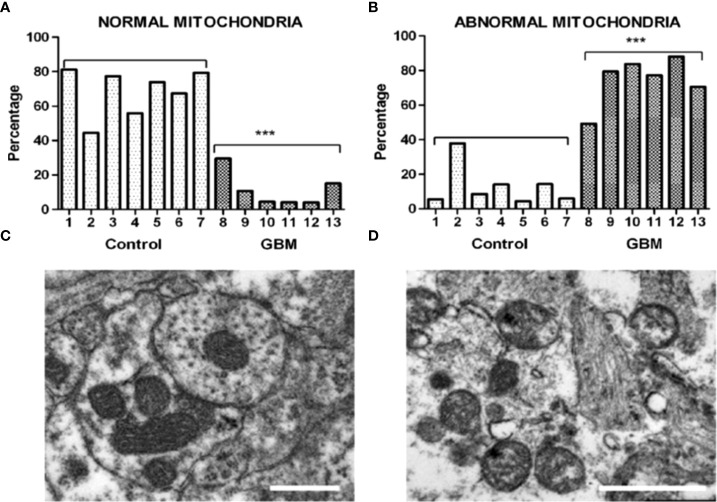
Morphological abnormalities seen in GBM mitochondria from the work of Deighton et al. (15). The morphology of 150 mitochondria was assessed in six GBM samples and in seven peri-tumoral control samples using Electron Microscopy (EM). **(A)** Percentage of normal mitochondria where cristae were visible throughout the mitochondria in peri-tumoral control and GBM samples (each bar represents one sample; *** p-value = 0.0001); **(B)** Percentage of abnormal mitochondria where cristae were sparse and abnormal in peri-tumoral control and GBM samples; *** p-value = 0.0001). **(C, D)** Representative EM images of normal and abnormal mitochondria, respectively. Cristolysis was significantly greater in mitochondria from GBM tissue than in mitochondria from normal surrounding brain tissue. The scale bars represent 0.5 um. The authors reported 117 mitochondrial proteins altered in GBM in association with ultrastructural mitochondrial abnormalities, similar to those described previously by Arismendi-Morillo et al. (16). ATP synthesis through OxPhos cannot be normal in tumor cells with these abnormalities. Image reproduced under a Creative Commons license from Deighton et al. (15).

GBM, like most major cancers, is dependent on fermentation metabolism for the synthesis of biomass and energy (ATP) regardless of the cellular or the genetic heterogeneity observed within the tumor (13, 14). A dependency on fermentation metabolism is the consequence of the well-documented abnormalities in the number, the structure, and the function of GBM mitochondria and mitochondrial associated membranes (MAM) and shown in **Figure 2**, and as described previously in detail (7, 14, 15, 17–25). In light of these structural and functional abnormalities, it would not be possible for GBM mitochondria to synthesize much if any ATP through OxPhos based on the foundational principle in evolutionary biology that structure determines function (14, 26, 27). The numerous reports suggesting that OxPhos is either normal or not seriously impaired in GBM cells is inconsistent with this foundational principle (28–40). It is important to recognize that oxygen consumption is not a reliable marker for OxPhos function in cancer cells (see below). It is unlikely that ATP synthesis through OxPhos could be normal in GBM cells that have documented abnormalities in mitochondria ultrastructure and function. Moreover, the large numbers of somatic mutations seen in GBM and in many other cancers, for that matter, arise as down-stream effects of OxPhos dysfunction with consequent ROS production (12). The somatic mutations in tumor cells will prevent adaptive versatility according to the evolutionary concepts of Darwin and Potts, thus locking in a dependency on fermentation metabolism for growth (41–44). It should be known, especially in the oncology field, that nothing in either general biology or in cancer biology can make sense except in the light of evolution (12, 45).

It is important to emphasize that a reduction in OxPhos of ~50% would dissipate the protonmotive force causing a reversal of the F_o_-F_1_ ATP synthase (7). The F_o_-F_1_ ATP synthase generally operates in forward mode (i.e., synthesizing ATP) only when the mitochondria are sufficiently polarized. The F_o_-F_1_ ATP synthase would be unable to generate ATP under a loss of electron transport chain operation on the order of 45-50% (7). This degree of loss would cause ATP hydrolysis, thus pumping protons out of the matrix. Reversal of the ATP synthase is what affords glutamine-driven mitochondrial substrate phosphorylation (mSLP) the critical role of providing ATP directly within the matrix when OxPhos becomes inhibited or impaired (7, 12). An inverse relationship between OxPhos efficiency and tumor aggression has been reported (46). A similar phenomenon has also been described with respect to the degree of fermentation and tumor growth, *i.e.*, the greater is the fermentation, the more aggressive is the cancer (14, 47–49). GBM cells, regardless of their cellular origin or genetic heterogeneity, are dependent on fermentation for survival due to abnormalities in mitochondrial structure and function.

A large part of the confusion on mitochondrial dysfunction in cancer comes from the incorrect assumption that oxygen consumption observed in cancer cells is linked to ATP synthesis through OxPhos (14, 28, 29, 40, 50–53). Many cancers, including GBM, can survive in hypoxia (0.1% oxygen) or in a solution of potassium cyanide, a Complex IV inhibitor, findings that would exclude normal OxPhos as a source of ATP synthesis (54–57). Cells with normal OxPhos function cannot survive for very long in cyanide or in hypoxia. While oxygen is necessary for cholesterol synthesis, GBM cells can obtain cholesterol from the microenvironment under hypoxic conditions (54, 58). Many normal cells and tumor cells will consume oxygen and ferment lactic acid when grown *in vitro*, but only tumor cells continue to ferment when grown *in vivo* (14, 47, 48).

The oxygen consumption in tumor cells is uncoupled and is used more for ROS production than for ATP synthesis through OxPhos (14, 23, 59–61). High-resolution oxygen consumption measurements and extracellular flux analysis, such as produced by Seahorse XF technology, cannot accurately measure OxPhos-driven ATP synthesis (53). Moreover, these measurements are highly variable in inter-laboratory settings (cell lines with exactly the same genetic background can display opposite metabolic profiles), are extrapolated using general, non-cancer specific ATP/O stoichiometries, and are limited by non-physiological and artefactual cell culture conditions (53, 62). It is not clear if most investigators using general purpose respirometry are aware of these facts.

Also contributing to misinformation on oxygen consumption and ATP synthesis is the failure to recognize glutamine-driven mSLP as a major source of energy for GBM cells (7, 14). Warburg was also unaware of this linkage, as he assumed that oxygen consumption was linked to OxPhos in his cancer cell preparations (7, 47, 48). Viewed collectively, these findings indicate that oxygen consumption alone cannot be used as a measure of OxPhos-derived ATP synthesis in most tumor cells including GBM.

## Mitochondrial substrate level phosphorylation drives ATP synthesis and microenvironment acidification in GBM

Recent studies have described how mSLP at the succinyl CoA synthetase reaction in the glutaminolysis pathway can provision ATP synthesis in GBM (7, 14, 53, 63, 64). The glutamine nitrogen produced from glutaminolysis is essential for the synthesis of nucleotides and amino acids. The waste products of glutaminolysis (primarily glutamic acid and succinic acid) would also contribute to acidification of the GBM microenvironment (7, 14, 65, 66). The catabolism of glutamine towards succinate will generate CO_2_ from the oxidative decarboxylation of the alpha-ketoglutarate dehydrogenation complex thus further acidifying the microenvironment. Additionally, succinate can stimulate NF-κB-driven inflammation and facilitate Hif-1α-driven glycolysis (7, 65, 67, 68). While the energetic competence of mitochondria in GBM and most other cancers is compromised in producing ATP through OxPhos, these mitochondria remain functional for other biosynthetic roles and in producing sufficient ATP through mSLP. Unlike OxPhos, however, that produces water and CO_2_ as waste products, mSLP produces glutamic acid and succinate acid as waste products that contribute to microenvironment acidification.

## Fermentation metabolites acidify the GBM microenvironment

The metabolic waste products of glucose and glutamine fermentation (lactic acid, glutamic acid, and succinic acid) will together acidify the GBM microenvironment. This acidification is ultimately responsible for drug resistance, enhanced invasion, immunosuppression, and metastasis (7, 14, 53, 69). Glucose carbons are essential for biomass synthesis through the glycolysis and the pentose phosphate pathways, with lactic acid and nucleic acid precursors produced as major end products (14, 70, 71). The pyruvate kinase M2 (PKM2) isoform, which is abundantly expressed in most malignant cancers, produces pyruvate-derived lactic acid with minimal ATP synthesis (14, 72–75). In other words, most of the glucose-derived lactic acid coming from the tumor cells is produced with little ATP synthesis through glycolysis. Some of the lactate acid produced in cancer cells can be returned to the tumor as glucose through the Cori cycle thus maintaining a constant supply of glucose to the tumor (76).

**Figure 3:**
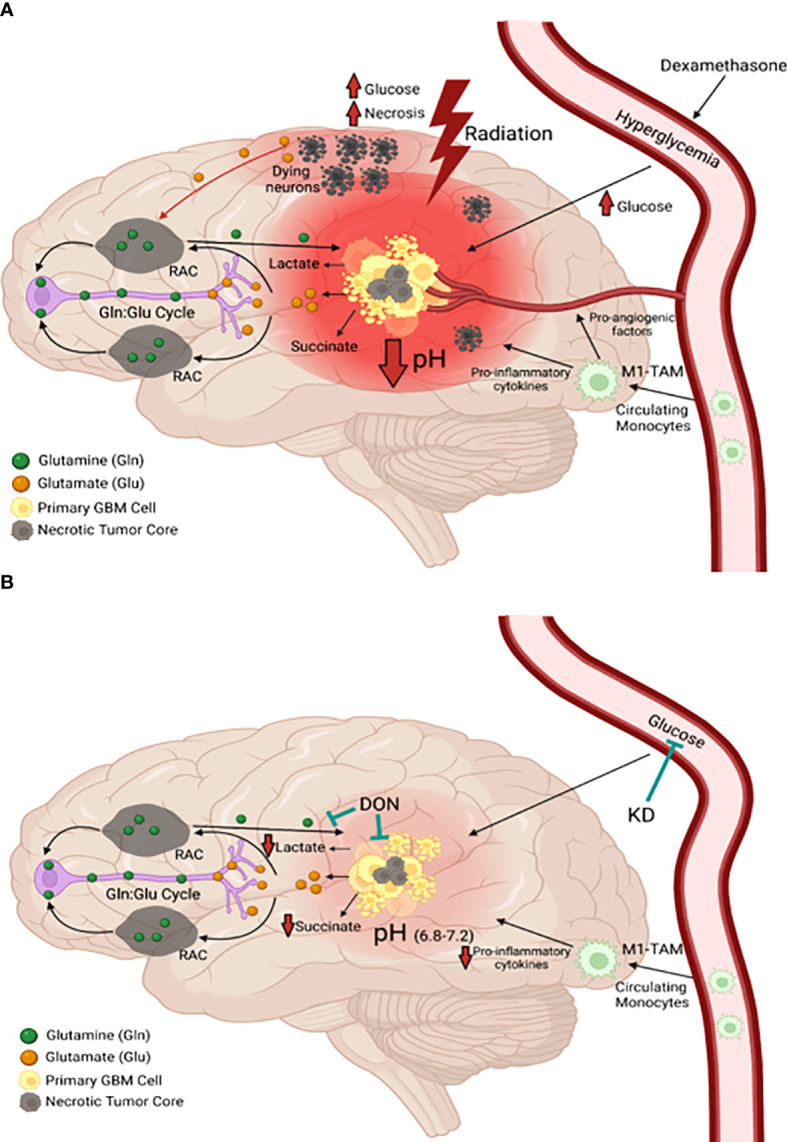
Origin and management of microenvironment acidity in GBM. **(A)** Glucose and glutamine are the primary energy metabolites necessary for driving rapid GBM growth. Glucose is the metabolic fuel necessary for nearly all brain functions under normal physiological conditions and is the major source of carbons for biomass synthesis through the glycolytic and pentose phosphate pathways in tumor cells. Tumor cells metabolize glutamine to glutamate, which is then metabolized to alpha-ketoglutarate. Significant energy is generated from the succinyl CoA ligase reaction (substrate level phosphorylation) in the glutaminolysis pathway using alpha-ketoglutarate-derived succinyl CoA as substrate (see **Figure 7**). In contrast to extracranial tissues, where glutamine is the most available amino acid, glutamine is tightly regulated in the brain through its involvement in the glutamate-glutamine cycle of neurotransmission (1, 78, 79). Glutamate is a major excitatory neurotransmitter that must be cleared rapidly following synaptic release in order to prevent excitotoxic damage to neurons (1, 79–81). Glial cells possess transporters for the clearance of extracellular glutamate, which is then metabolized to glutamine for delivery back to neurons. Neurons metabolize the glutamine to glutamate, which is then repackaged into synaptic vesicles for synaptic release (1). This cycle maintains low extracellular levels of both glutamate and glutamine in the normal neural parenchyma. Disruption of the cycle can provide neoplastic GBM cells access to glutamine. Besides serving as a metabolic fuel for the neoplastic tumor cells, glutamine is also an important fuel for cells of myeloid linage, which include macrophages, monocytes, microglia, and especially the invasive mesenchymal cells in GBM (1, 13, 82–84). In contrast to the proliferative GBM stem cells, the neoplastic GBM mesenchymal cells are thought to be derived from microglia or from microglia-stem cell fusion hybrids, which would have immuno-suppressive properties (82, 85). As long as GBM cells have access to glucose and glutamine, the tumor will grow and acidify the microenvironment making long-term management difficult. The current treatments for GBM (radiation and TMZ chemotherapy) will further increase glucose and glutamine availability, creating an unnecessary metabolic storm that will enhance microenvironment acidification and rapid tumor recurrence. The red hue is indicative of the inflammation and acidification of the tumor microenvironment (see text for further details). **(B)** The simultaneous restriction of glucose and glutamine, while elevating non-fermentable, anti-inflammatory ketone bodies, will reduce acidification, restore the pH balance of the microenvironment, and growth arrest or kill most of the neoplastic cells (11–13). RAC, reactive astrocytes; TAM, tumor-associated macrophages; Gln, glutamine; Glu, glutamate. These images were modified from that in (86).

Calorie restriction, which lowers blood glucose and elevates blood D-β-OHB, reduces nuclear expression of phosphorylated NF-κB (p65), cytosolic expression of phosphorylated IκB, total IκB, and DNA promoter binding activity of activated NF-κB in the CT-2A astrocytoma (77). NF-κB is a major driver of inflammation in the GBM microenvironment. **Figure 3A** shows how the waste products of glucose and glutamine fermentation are largely responsible for the inflammation and acidification in the GBM microenvironment. Hence, therapies that can lower blood glucose while elevating D-β-OHB will mitigate microenvironment inflammation and acidification through multiple mechanisms.

## Current therapies could enhance microenvironment acidity and recurrence of GBM

The current treatment for GBM management involves debulking surgery, radiotherapy, and temozolomide chemotherapy (TMZ) (1, 8, 9, 87). While the waste products of glucose and glutamine fermentation will contribute to microenvironment acidification and the rapid growth of untreated GBM, the current treatments used for GBM management could also accelerate these processes after a growth delay following surgical debulking (1, 88, 89). It is documented that radiotherapy produces significant necrosis and hypoxia in the tumor microenvironment (1, 90–92). Radiotherapy disrupts the tightly regulated glutamine-glutamate cycle in the neural parenchyma thus increasing the levels of glutamine and glutamic acid as described further in **Figure 3A**.

Glutamic acid is an excitotoxic amino acid that enhances GBM invasion (1, 80, 81, 86, 93–96). Radiotherapy also damages the brain microenvironment, which increases glucose and glutamine availability to the tumor cells thus driving tumor growth through hyperglycolysis, necrosis, and acidification. While chemo-radiotherapy might have a role in the treatment of low-grade non-neural tumors, these confounding variables are ultimately responsible for GBM therapy resistance (1, 90, 97–99).

## Blood glucose is linked to rapid GBM growth

**Figure 4:**
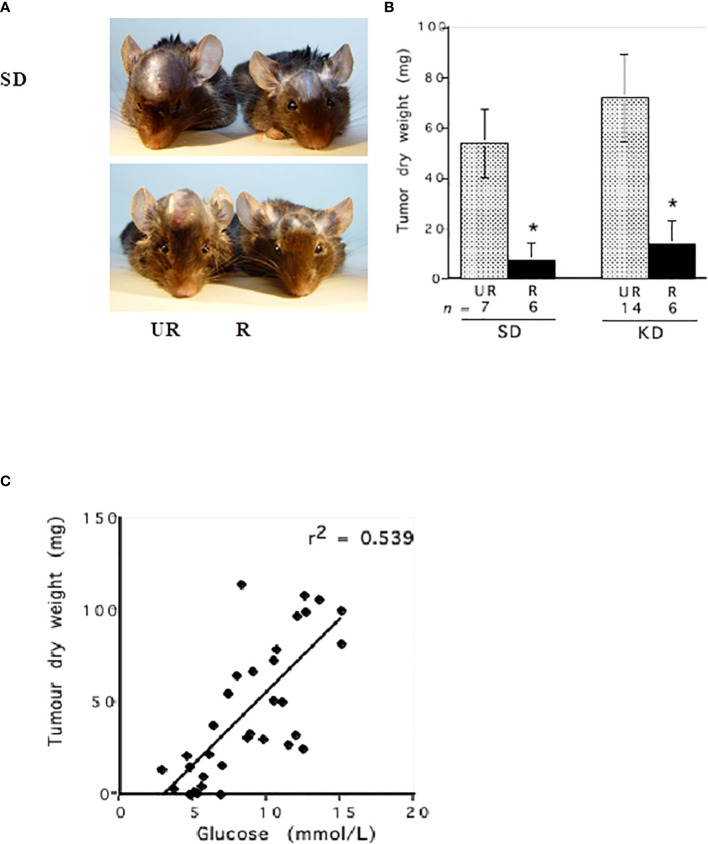
Influence of diet on the intracerebral growth of the CT-2A malignant astrocytoma. Dietary treatment was initiated 1 day after tumor implantation and was continued for 13 days. The visual representation **(A)** and quantitative assessment **(B)** of tumor growth in C57BL/6J mice receiving either the standard diet (SD) or ketogenic diet (KD) under either unrestricted (UR) or restricted (R) feeding. The asterisk indicates that the dry weights of the tumours in R groups were significantly lower than those in the UR groups at P < 0.01. **(C)** Linear regression analysis of plasma glucose and CT-2A-tumor growth in mice from both the SD and KD dietary groups combined (n = 34). These analyses included the values for plasma glucose and tumor growth of individual mice from both the UR and R-fed groups. The linear regression was highly significant at P < 0.001, indicating that blood glucose levels predict CT-2A tumor growth rate (100). The failure of the KD-UR to reduce blood glucose levels and tumor growth could be due to insulin insensitivity in this mouse strain (102). Images reproduced under a Creative Commons license.

**Figure 5:**
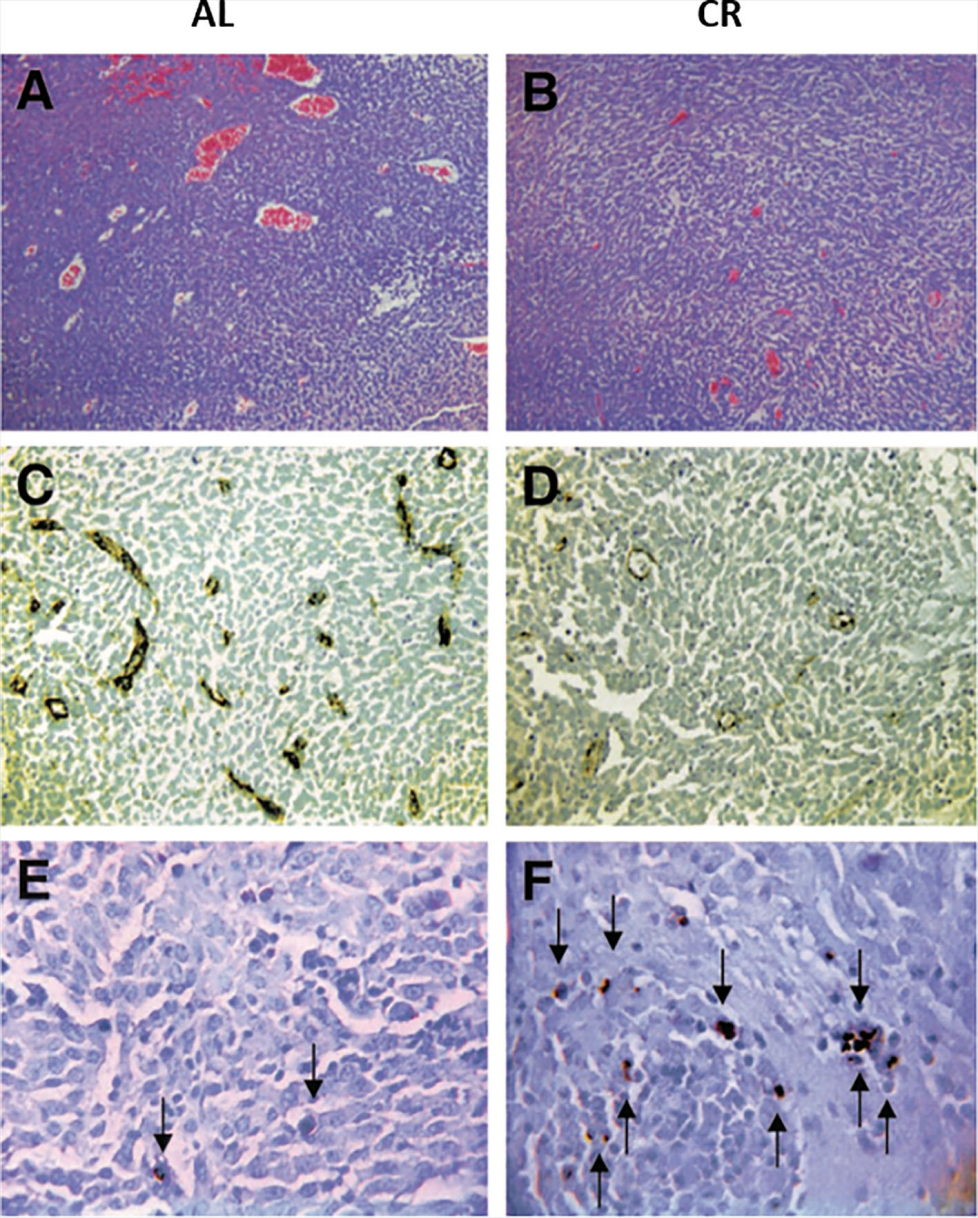
Influence of calorie restriction (CR) on microvessel density and apoptosis in the CT-2A malignant astrocytoma. CR was initiated 7 days before intracerebral tumor implantation and was continued for 11 days. H & E stained tumor sections in an *ad libitum* (AL) mouse **(A)** and in a CR mouse **(B)** (100X). Factor VIII immunostaining from the tumor grown in an AL mouse **(C)** and in a CR mouse **(D)** (200X). TUNEL positive apoptotic cells (arrows) from the tumor grown in an AL mouse **(E)** and in a CR mouse **(F)** (400X). Each stained section was representative of the entire tumor. All images were produced from digital photography. Image reproduced under a Creative Commons license from (119).

Linear regression analysis showed that blood glucose could predict the growth rate of the CT-2A malignant mouse astrocytoma, a stem-cell tumor (100, 101) (**Figures 4A–C**). Evidence also shows that survival is lower in GBM patients with higher blood glucose levels than in GBM patients with lower glucose levels (1, 103–111). Although the dexamethasone steroid is often prescribed along with standard treatments to reduce vasogenic edema, steroids will elevate blood glucose levels thus contributing indirectly to tumor growth (1, 112–114). Alternatives to dexamethasone for reducing vasogenic edema should receive consideration (115). Radiotherapy also increases blood glucose levels and facilitates hybridizations between tumor cells and macrophage/microglia thus producing highly invasive metastatic cells (1, 82, 108, 116–118). As glucose-derived lactic acid is the end product of glycolysis, GBM treatments that would elevate blood glucose levels will contribute to elevated lactic acid, microenvironment acidification, and tumor recurrence. Conversely, therapeutic strategies that would reduce glucose levels will lower lactic acid production, microenvironment acidification, and tumor recurrence (**Figure 3B**). It is clear from **Figure 5** that calorie restriction, which lowers blood glucose while elevating ketone bodies, reduces microvessel density (angiogenesis) and increases tumor cell apoptosis in the CT-2A malignant astrocytoma. Hence, the dietary restriction of blood glucose can reduce microenvironment acidification through therapeutic effects on inflammation, angiogenesis and apoptosis.

## Mesenchymal cells will contribute to GBM acidification

Accumulating evidence shows that the highly invasive mesenchymal cells seen in GBM are derived from neoplastic microglia or from microglia/macrophages that hybridize with non-invasive cancer stem cells, similar to that reported for other highly invasive metastatic cancers (82, 118, 120–124). Indeed, up to 60% of the cells in some GBM contain macrophage characteristics (125–128). We described how the neoplastic GBM cells with mesenchymal characteristics can be derived from transformed macrophages/microglia (13, 82, 129–131). As activated macrophages are immunosuppressive and acidify the microenvironment, it should be no surprise why immunotherapies have been largely ineffective in managing GBM (1, 132–134). The mesenchymal cells seen in GBM, whether part of the neoplastic cell population or part of the infiltrating cell population, will acidify the microenvironment through a variety of inflammation-linked mechanisms (135, 136). Some researchers also consider tumor cell-derived lactate as a checkpoint due to its ability to block immunotherapies (69). As lactate is derived from glucose, glucose restriction should reduce this “so called” checkpoint inhibitor. Hence, the mesenchymal cell populations in GBM will not only contribute to microenvironment acidification, but will also contribute to their own survival using glutamine as a metabolic fuel (13, 137, 138).

## Can metabolic therapy improve immunotherapy?

Immunotherapies have not yet been effective GBM management, but could be effective if there is evidence showing that they will not increase availability of glucose and glutamine in the tumor microenvironment, enhance inflammation, or cause hyper-progressive disease, as was documented in non-small cell lung cancer (139). Inflammatory oncotaxis, arising from surgical resection or from biopsy of lower-grade brain tumors, could also contribute to the transformation to high-grade secondary GBM (140–143). As the neoplastic macrophage/mesenchymal cells seen in GBM are dependent to a large degree on glutamine (13, 144), glutamine restriction will be essential for targeting these cells as we recently demonstrated (13). Recent studies show that a ketogenic diet can enhance the efficacy of immunotherapy (145). Most importantly, the simultaneous restriction of glucose and glutamine could improve the therapeutic efficacy of immunotherapies.

## GBM chemotherapy can contribute to microenvironment acidification

TMZ chemotherapy can contribute to microenvironment acidification through adverse effects on mitochondrial OxPhos function and increased production of GBM driver mutations (1, 9, 146, 147). In addition to increasing blood glucose levels, dexamethasone also increases glutamine levels through its induction of glutamine synthetase activity (7, 11, 86, 113, 148, 149). Bevacizumab (Avastin) is also widely prescribed to GBM patients to reduce angiogenesis (150–152). Bevacizumab, however, increases tumor necrosis while selecting for the most invasive and therapy-resistant tumor cells (153, 154). As both bevacizumab and TMZ damage mitochondria (155), these drugs will contribute further to tumor cell reliance on fermentation metabolism thus increasing microenvironment acidification (7, 11). Considered together, the current GBM chemotherapies inflict damage to the microenvironment and facilitate availability of glucose and glutamine to the neoplastic tumor cells, all of which will contribute to tumor recurrence, further acidification, and rapid progression (**Figure 3A**). It is not likely that overall patient survival could be improved when using therapies that increase distal tumor cell invasion and microenvironment acidification.

It should also be recognized that human cytomegalovirus (HCMV) infects many GBM that would further facilitate tumor cell use of glutamine and glucose (1, 156, 157). Recent studies show that vaccine-targeting of the HCMV pp65 protein could increase progression free and overall survival of some GBM patients (158). It would be interesting to determine if this therapeutic effect resulted in part from inhibition of the glycolysis or the glutaminolysis pathways in GBM cells (159, 160). Glucose and glutamine are required for synthesis of glutathione while glutamine is essential for the action of manganese superoxide dismutase (161–163). Consequently, the elevated use of glucose and glutamine, which increases anti-oxidant potential, will contribute to the resistance of GBM cells to chemotherapy and radiotherapy.

It is known that elevated aerobic fermentation (Warburg effect) also drives the multidrug resistant (MDR) phenotype, which protects GBM cells from toxic chemotherapy (1, 5, 41, 164). Hence, the treatment-linked increases in fermentable energy metabolites and disruption of the tumor microenvironment can explain in large part how overall survival remains so poor for most GBM patients treated with current standard of care (7, 97). The information presented in **Figure 3A** describes how current therapies can facilitate rapid GBM recurrence. It is our view that these therapies can account in large part the remarkable reproducibility of poor patient survival across multiple surgical institutions as seen in **Figure 1**. It is unlikely that GBM patient survival will improve significantly if therapies that increase microenvironment acidification and are inherently ineffective are continuously used (165).

## Ketone bodies are non-fermentable and can reduce GBM acidification

As fermentation metabolism is ultimately responsible for rapid GBM growth and the acidification of the microenvironment, then therapies that target fermentation metabolism should reduce acidification and GBM growth. Metabolic therapy involves diet/drug combinations that target the availability of glucose and glutamine while also elevating non-fermentable, anti-inflammatory ketone bodies (13, 41, 166–170). Most importantly, GBM and other tumors cannot use ketone bodies for energy due to deficiencies in SCOT; the key mitochondrial enzyme needed for ketone body metabolism (171–173). No evidence has been presented, to our knowledge, showing that ketone bodies can replace glucose or glutamine in serum free media for the survival of any tumor cell. Ketogenic diets and water-only therapeutic fasting will lower circulating glucose levels while elevating circulating D-β-hydroxybutyrate (D-β-OHB) levels (41, 174–176). Water-only fasting in humans is comparable to a 40% calorie restriction in mice due to differences in basal metabolic rate that about six times faster in mice than in humans (177). Therapeutic strategies that lower blood glucose while elevating blood ketone bodies are anti-angiogenic, anti-edematous, anti-inflammatory, and pro-apoptotic. Evidence supporting this statement was described previously (13, 178). Diets that lower glucose and elevate D-β-OHB can also reduce circulating levels of insulin-like growth factor 1 **(**IGF-1), a known driver of tumor growth **(** **Table 1**). There is no known drug that can produce the broad range of therapeutic effects as can diets that reduce glucose while elevating D-β-OHB.

**Table 1:** Influence of diet on plasma glucose, β-OHB, and IGF-I levels in mice bearing the CT-2A intracerebral brain tumour^a^.

Diet^b^	Groups^c^	Glucose (mmol I^-I^)	β-OHB (mmol I^-I^)	IGF-I (ng ml I^-I^)
SD	UR	9.1 ± 0.9	0.6 ± 0.1	208 ± 25
	R	( 7)^d^5.2 ± 1.1*	(7)1.4 ± 0.2*	(6)117 ± 36*
		(6 )	(6)	(6)
KD	UR	11.4 ± 1.4	1.0 ± 0 .3	294 ± 30
		(14)	(14)	(5 )
	R	5.7 ± 1.5*	1.3 ± 0.6	193 ± 57*
		(6)	(6)	(6)

**^a^** Values are expressed as means **±** 95% confidence intervals. **^b^** Animals were fed either a standard chow diet (SD) or a ketogenic diet (KD). **^c^** UR (unrestricted feeding) and R (restricted to 60% of the SD-UR group. **^d^** Numbers in parentheses indicate the number of independent tumor-bearing mice examined in each group. The asterisks indicate that the values of the R groups differed from those of their respective UR groups at P < 0.01. The details of these experiments are as we described (100).

It is important to mention that blood glucose can be reduced to very low levels (less than 1.0 mM) in humans that are in therapeutic ketosis (6-8 mM, D-β-OHB) without producing hypoglycemic reactions (174, 179, 180). A whole-body transition from glucose-driven metabolism to D-β-OHB-driven metabolism will reduce circulating glucose levels thus reducing extracellular acidification from lactic acid production. At the same time, this transition will also produce metabolic stress on all neoplastic GBM cells that are dependent on glycolysis for growth (41, 172, 174, 181). Moreover, D-β-OHB metabolism enhances the ΔG’ATP hydrolysis in normal cells from -56 kJ/mole to -59 kJ/mole, thus providing normal cells with an energetic advantage over the fermentation-dependent tumor cells (41). We are not familiar with any therapies, besides ketogenic metabolic therapy (KMT), that can enhance the energetic advantage of normal cells over that of tumor cells (11, 41).

The energetic advantage of D−β-OHB metabolism in normal cells is seen predominantly with D-β-OHB, and is not seen with either the D/L- β-OHB racemic mixture or with fatty acids (174, 182, 183). On the other hand, racemic D/L-β-OHB tends to reduce blood glucose more through shifting redox state in the liver and can potentially increase ROS production in tumor cells through β-oxidation of the L-form (41). The L-β-OHB interconverts back to D-β-OHB (in tissues) through a racemase enzyme or gets converted to acetyl-CoA. The L-β-OHB also has greater potential as a signaling molecule since it remains in circulation longer and has similar effects at suppressing the NLRP3 inflammasome and epigenetic effects (184–186). Hence, D-β-OHB and D/L-β-OHB can stress tumor cell metabolism while enhancing the metabolism of normal cells through a variety of mechanisms.

The therapeutic effects seen with ketone bodies are generally best when blood glucose levels are low (generally below 3.6 mM), as little or no therapeutic benefit is seen in either preclinical GBM models or in human patients when glucose levels remain elevated (100, 171, 187). These therapeutic glucose levels could be difficult to achieve for many GBM patients, however, due to the glucose-elevating effects of the current standard treatments used to manage GBM. We also did not find any therapeutic benefit of sodium bicarbonate on the growth of the VM-M3 mouse glioblastoma suggesting that alkalinization using sodium bicarbonate was ineffective in managing this GBM model (L. Shelton, unpublished). It is the synergistic action of low blood glucose with elevated ketone bodies that provides the best therapeutic strategy for slowing growth and reducing microenvironment inflammation and acidification.

## The simultaneous restriction of glutamine and glucose will reduce GBM growth and acidification

**Figure 6:**
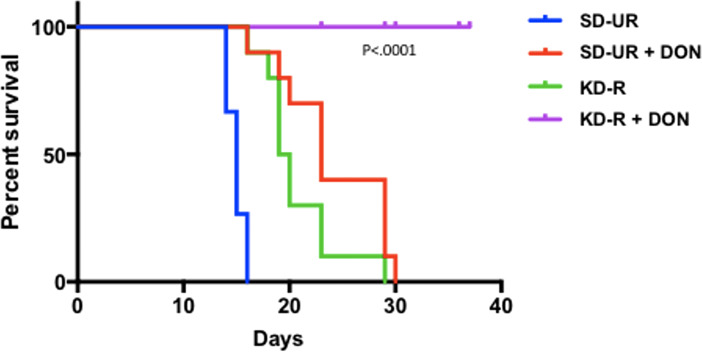
Influence of diet/drug therapy on overall survival of VM/Dk mice with the VM-M3 invasive GBM. A calorie restricted ketogenic diet (KD-R) was administered together with the glutaminase inhibitor, 6-diazo-5-oxo-L-norleucine (DON) as we described (13). Overall survival was significantly longer in the tumor bearing mice receiving the diet/drug combination (KD-R + DON) than in the mice receiving the standard high-carbohydrate diet (SD-UR), the KD-R alone, or DON alone. It is important to mention that 2-3x more DON was delivered to the tumor of the mice fed the KD-R than to the mice fed the SD-UR indicating that the KD facilitates a non-toxic delivery of small drug molecules through the blood brain barrier (13, 188). Image reproduced under a Creative Commons license from (13).

**Figure 7:**
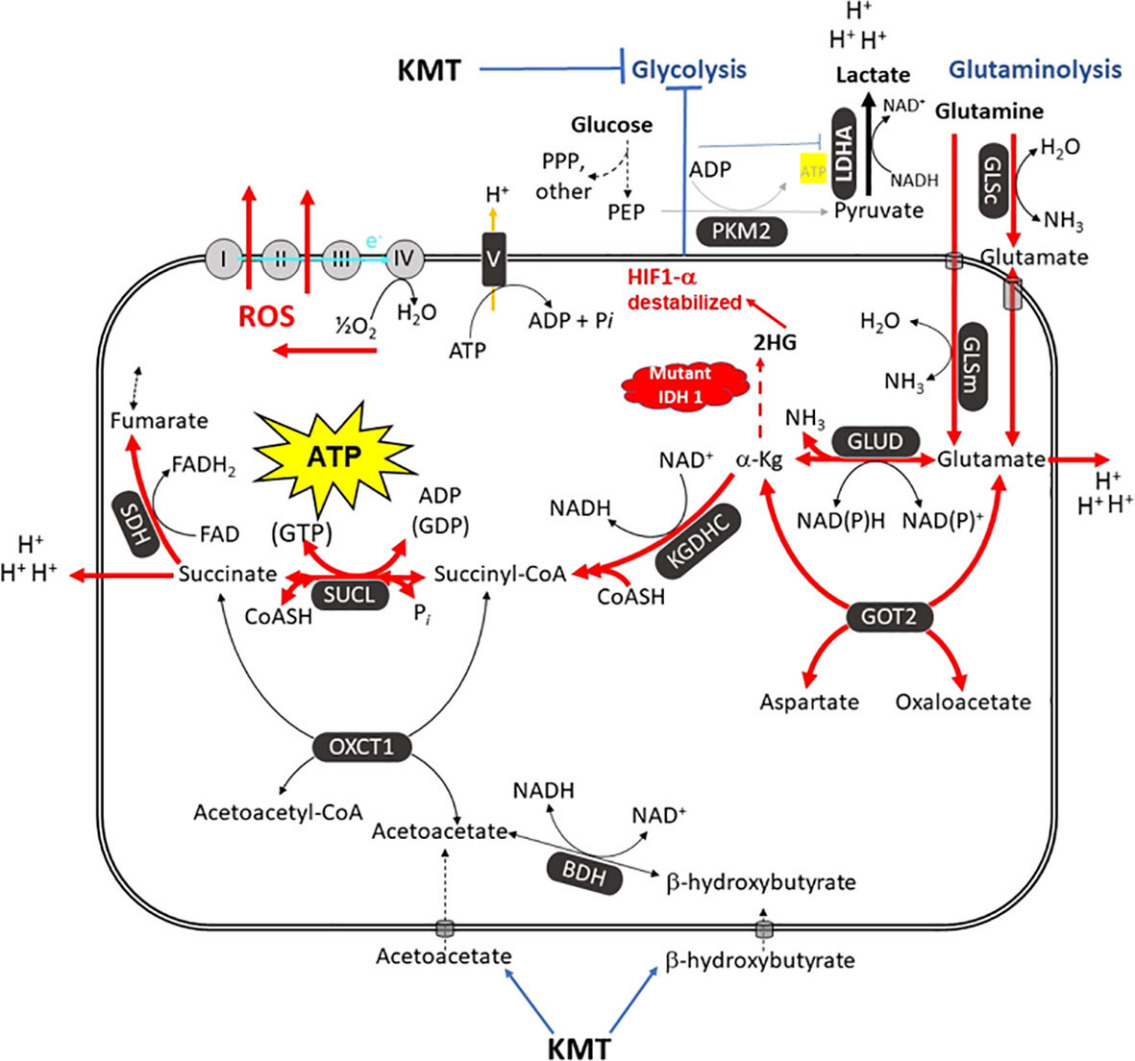
The metabolic pathways responsible for the acidification of the GBM microenvironment. GBM growth is dependent on glucose carbons for biomass synthesis through glycolysis and glutamine carbons for ATP synthesis through glutaminolysis. The glutamine nitrogen is necessary for protein and nucleic acid synthesis. The waste products of the glycolytic and the glutaminolysis pathways (lactic acid, glutamic acid, and succinic acid) will acidify the GBM microenvironment. The oxygen consumption is linked to ROS production, not to ATP synthesis. Excessive ROS produce somatic mutations and further increase inflammation and acidification of the microenvironment (7, 12, 14, 194). A calorie restricted KD will reduce glucose availability for glycolysis while also interfering with the glutaminolysis pathway (11). Glutamine-driven mSLP in the glutaminolysis pathway is a major source of ATP synthesis for GBM cells (7, 14). The glutaminolysis pathway (red) becomes dominant in tumor cells with inefficient OxPhos and that express the dimeric PKM2 isoform. PKM2 is expressed in GBM and produces less ATP through glycolysis than does the PKM1 isoform (73, 75, 195, 196). The elevation of ketone bodies (D-β-hydroxybutyrate and acetoacetate) through KD will indirectly reduce ATP synthesis through the succinate CoA ligase (SUCL) reaction by diverting CoA from succinate to acetoacetate. The *IDH1* mutation could reduce ATP synthesis through mSLP by increasing synthesis of 2-hydroxyglutarate that is derived from α-ketoglutarate and thus reduce the succinyl CoA substrate for the SUCL reaction (11, 14, 197). Besides its potential effect in reducing glutaminolysis, 2-hydroxyglutarate can also target multiple HIF1α-responsive genes and enzymes in the glycolysis pathway thus limiting synthesis of metabolites and one-carbon metabolism needed for rapid tumor growth (7, 14, 68, 198). The down regulation of Hif1-α-regulated lactate dehydrogenase A (LDHA), through the action of both the KD and the *IDH1* mutation, will reduce extracellular lactate levels thus further reducing microenvironment inflammation, acidification, and tumor cell invasion. Hence, the simultaneous inhibition of glycolysis and glutaminolysis through the synergistic effects KMT and the *IDH1* mutation will reduce the majority of signaling pathways necessary for rapid GBM growth and acidification of the microenvironment. BDH, β-hydroxybutyrate dehydrogenase; FAD, flavin adenine dinucleotide; GLSc, glutaminase cytosolic; GLSm, glutaminase mitochondrial; GLUD, glutamate dehydrogenase; GOT2, aspartate aminotransferase; KGDHC, α-ketoglutarate dehydrogenase complex; LDHA, lactate dehydrogenase A; NME, nucleoside diphosphate kinase; OXCT1, succinyl-CoA:3-ketoacid coenzyme A transferase 1; PC, pyruvate carboxylase; PDH, pyruvate dehydrogenase; PEP, phosphoenolpyruvate; PKM2, pyruvate kinase M2; SDH, succinate dehydrogenase; SUCL, succinate-CoA ligase. **KMT,** Ketogenic metabolic therapy. Reprinted with modifications from (14).

In addition to glucose, glutamine is the other major fuel that drives GBM growth especially the neoplastic mesenchymal cells (14, 137, 144). We showed that the glutamine-targeting analogue, 6-diazo-5-oxo-L-norleucine (DON), used with a calorie restricted ketogenic diet could significantly reduce growth and improve overall survival in preclinical models of GBM (**Figure 6**). Moreover, we found that ketogenic diets facilitated delivery of DON and other small molecules through the blood brain barrier (13, 188). This delivery may be due in part to the action of the content of caprylic acid in the diet (189). Hyperbaric oxygen therapy can also reduce angiogenesis and microenvironment inflammation especially in combination with therapeutic ketosis (41, 190–193). In addition to findings in preclinical models, we also described how the *IDH1* mutation could act as a therapeutic drug that simultaneous targets the glycolysis and glutaminolysis pathways to improve survival in a GBM patient (11) (**Figure 7**). The long term survival of this patient, now at eight years, was attributed to a combination of his younger age, his low-carbohydrate ketogenic diet, his acquired *IDH1* mutation, and finally to his avoidance of radiation, TMZ, and steroids (11). Ketogenic metabolic therapy involves the synergistic therapeutic action of the KD used with drugs and procedures that restrict availability of glucose and glutamine while providing normal cells with an energetic advantage over tumor cells that are limited to energy generation through fermentation (12, 41). More recent studies also support some of these observations in younger GBM patients (199). Persistent statements suggesting that tumor cells have a growth advantage over normal cells make no sense in the light of evolutionary theory (12). The metabolic pathways contributing to GBM microenvironment acidification and their management by KMT and the *IDH1* mutation are described in **Figure 7**.

## Limitations

There are several limitations that currently prevent the application of metabolic therapy for reducing microenvironment acidification and the growth of GBM. First, the dosage, timing, and scheduling of the diet/drug combinations that can best target glucose and glutamine availability have yet to be optimized for most GBM patients (1, 11, 41, 87). Second, the findings that GBM is largely dependent on glucose and glutamine fermentation for growth due to OxPhos deficiency is inconsistent with the current dogmatic view that GBM and most other cancers are exceedingly complex genetic diseases requiring complicated Rube Goldberg-type solutions (12). Finally, the most important limitation for adapting metabolic therapy in the clinic is the absence of a business model that can generate sufficient replacement revenue using cost-effective, non-toxic metabolic therapies (200–203). We predict that major advances in overall GBM patient survival will be realized once GBM becomes recognized as a mitochondrial metabolic disease and when non-toxic metabolic therapies become the standard of care for management.

## Conclusions

Microenvironment acidification is largely responsible for drug resistance, enhanced invasion, immunosuppression, and metastasis. The acidic waste products of glucose and glutamine fermentation metabolism (lactic acid, glutamic acid, and succinic acid), generated within the neoplastic tumor cells, are responsible for the acidification of the GBM microenvironment. Stated simply: The greater is the availability of fermentable fuels, the greater is the resistance to therapy. The cancer microenvironment will heal itself if the origin of the acidification can be removed. Therapeutic strategies that restrict the availability of fermentable fuels, while increasing levels of non-fermentable ketone bodies, will reduce acidification, eliminate the majority of neoplastic tumor cells, and thus improve GBM management.

## Author contributions

All authors read and approved the information presented in the manuscript. ST wrote the manuscript. A-MG, ZG, LD, DT, EA, MJ, MP, TL, D’AD, KM, and CC made material contributions to content and revisions.

## Funding

We thank the Foundation for Metabolic Cancer Therapies, CrossFit Inc., The Nelson and Claudia Peltz Family Foundation, Lewis Topper, The John and Kathy Garcia Foundation, Mr. Edward Miller, Kenneth Rainin Foundation, the Corkin Family Foundation, Children with Cancer UK, and the Boston College Research Expense Fund for their support. The funders had no role in study design, data collection and analysis, decision to publish, or preparation of the manuscript.

## Conflict of interest

Author KM was employed by the company BERG LLC. Author SL was employed by the company Matterworks.

The remaining authors declare that the research was conducted in the absence of any commercial or financial relationships that could be construed as the potential conflict of interest. 

## Publisher’s note

All claims expressed in this article are solely those of the authors and do not necessarily represent those of their affiliated organizations, or those of the publisher, the editors and the reviewers. Any product that may be evaluated in this article, or claim that may be made by its manufacturer, is not guaranteed or endorsed by the publisher.

## Abbreviations

PPP, pentose phosphate pathway; OxPhos, oxidative phosphorylation; mSLP, mitochondrial substrate level phosphorylation; TMZ, temozolomide; D-β-OHB, D-β-hydroxybutyrate; KD, ketogenic diet; KMT, Ketogenic metabolic therapy; ROS, reactive oxygen species.
